# Molecular dynamics analysis of elastic properties and new phase formation during amorphous ices transformations

**DOI:** 10.1038/s41598-022-17666-2

**Published:** 2022-08-03

**Authors:** Anastasiia Garkul, Vladimir Stegailov

**Affiliations:** 1 Joint Institute for High Temperatures of the Russian Academy of Sciences, 125412 Moscow, Russia; 2 Moscow Institute of Physics and Technology (National Research University), 141701 Dolgoprudny, Russia; 3 National Research University Higher School of Economics, 109028 Moscow, Russia

**Keywords:** Atomistic models, Phase transitions and critical phenomena

## Abstract

Unlike conventional first-order phase transitions, the kinetics of amorphous-amorphous transitions has been much less studied. The ultrasonic experiments on the transformations between low-density and high-density amorphous ice induced by pressure or heating provided the pressure and temperature dependencies of elastic moduli. In this article, we make an attempt to build a microscopic picture of these experimentally studied transformations using the molecular dynamics method with the TIP4P/Ice water model. We study carefully the dependence of the results of elastic constants calculations on the deformation rates. The system size effects are considered as well. The comparison with the experimental data enriches our understanding of the transitions observed. Our modeling gives new information about the formation mechanisms of new phase clusters during the transition between low-density and high-density amorphous ices. We analyse the applicability of the term “nucleation” for these processes.

## Introduction

Water is central to many physical, biological and industrial applications and exhibits physical properties that are qualitatively different from those of most other liquids. Despite the huge amount of theoretical and experimental research aimed at studying the many specific properties of water, it continues to be one of the most mysterious pure substances in the Universe. Supercooled water, known for its polymorphism, attracts a lot of interest. Today the phase diagram of water boasts no less than 19 modifications^1^ of crystalline ice and 3 amorphous forms (not counting their derivatives)^2^, the most discussed of them are the low density amorphous ice (LDA) and the high density amorphous ice (HDA). Amorphous ice practically does not occur on Earth in nature, but LDA is the most widespread form of $\hbox {H}_2\hbox {O}$ in the Universe, as it is a part of interstellar dust and comet nuclei. LDA can be obtained from the gas phase^3^ or from liquid water^4^.

Actually, the discovery by Mishima et al.^5^ of solid-state amorphization (SSA) Ih $\rightarrow$ HDA in 1984 launched the entire epoch of the studies of the nature of these non-equilibrium transformations. It is noteworthy that amorphous forms of water were obtained from stable phases of all three states of aggregation^6^. Under isothermal compression of LDA or of hexagonal ice Ih, a sharp transition occurs, which leads to the formation of HDA, and LDA can then be restored from HDA under isothermal decompression.

**Figure 1:**
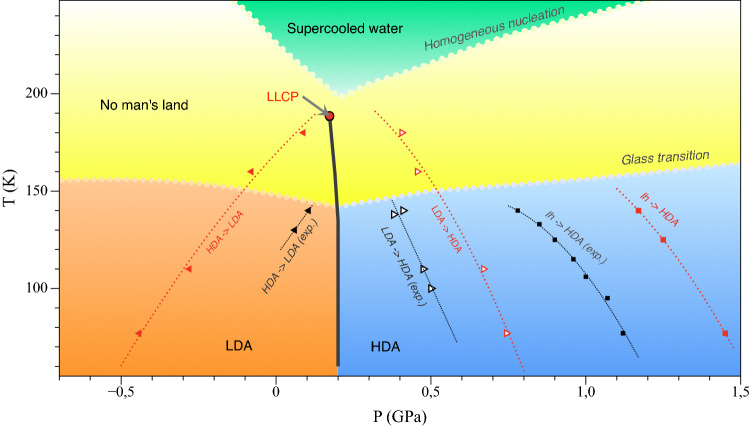
The T-P diagram of amorphous and liquid water. The separation line between a low density liquid and a high density liquid ends at the liquid–liquid critical point (LLCP)^17^. This point is located in an area called “no man’s land”, marked in yellow, where only crystalline forms of ice are experimentally observed. Transition lines based on the experiment^30^ are indicated by black dotted lines; the lines based on the results of this work for the TIP4P/Ice model are in red dotted lines (the corresponding compression/decompression rate of 0.1 GPa/ns).

An important question about the nature of amorphous ices is the hypothesis of the two-liquid model of water and the existence of a second critical point. One^7^ of several^7–9^ theoretical scenarios to explain water anomalies (such as a sharp increase in its isothermal compressibility, isobaric heat capacity, coefficient of the thermal expansion upon cooling below the equilibrium freezing point) postulates the existence of a discontinuous (first-order-like) transition between two forms of liquid water that correspond to LDA and HDA below the glass transition line (Fig. 1) and ends at the liquid-liquid critical point (LLCP). It is assumed that LLCP is located in the area called the “no man’s land” corresponding to the temperature range of $\sim$150–220 K, where only crystalline forms of water are observed in experiments due to very rapid crystallization. With all the abundance of experimental observations^10–15^ that indirectly indicate the possibility of the existence of the liquid-liquid transition in supercooled water, not a single unambiguous experiment has proved this thesis yet. Experimental problems under these conditions are associated with the inevitable rapid crystallization of a metastable liquid state, although the recent study by Kim et al.^16^ provides a convincing evidence.

Molecular modelling plays an important role in studying the behavior of water under such conditions^17–21^. It allows heating at sufficiently high rates to avoid crystallization and at the same time to study the structure in detail. Moreover, some water models demonstrate the liquid–liquid transition^22–24^, others do not^25^. The TIP4P class of water models provides a well balanced instrument that allows predictive modelling of water-based systems at reasonable computational expenses and, therefore, at relatively large length and time scales (see e.g.^26–29^).

Various approaches are used to study structure of the amorphous ices: an analysis of partial radial distribution functions (RDFs)^31,32^, the vibrational properties^33^, the hydrogen bond network^21^, the dynamical order parameter^34^ and classification using neural networks^35^. In this work, we rely on the partial RDFs O–O and on the distribution of angles between hydrogen-bonded oxygen atoms that reflects the order in the tetrahedral structure. It was shown^33^ that the tetrahedral structure is distorted in the Ih $\rightarrow$ LDA $\rightarrow$ HDA $\rightarrow$ liquid water sequence.

One of the tasks of this work is to form a microscopic understanding of the close relationship between elastic properties and the nature of a dynamic disorder present in amorphous ices. Knowledge of elastic properties combined with direct density measurements in amorphous ices is very useful for understanding their transformation mechanisms. The three-stage scenario of the heating induced transformation HDA $\rightarrow$ LDA discovered by Gromnitskaya et al.^30^ clearly demonstrates complex kinetics in amorphous networks, which indicates the complex nature of glassy water. It was also shown that elastic softening precedes amorphous-amorphous and crystal-amorphous transformations. But the theoretical interpretation of these results is still absent and has not been considered in any molecular modelling studies yet.

## Methods

### Simulation details

In this work, we consider classical molecular dynamics modelling of amorphous ice using the TIP4P/Ice water model^36^ that, for example, together with the TIP4P/2005 variant have been recently used for LLCP calculations^17^ and for supercooled and amorphous water studies^19,37,38^. Positions of particles are determined from the solution of the classical Newtonian equations of motion. The simulations are carried out for a system of $N=$ 2880, $N=$ 23040 and $N=$ 77760 water molecules, in a cubic box with periodic boundary conditions using the LAMMPS software package^39^ with the GPU acceleration for TIP4P models^40,41^. The cutoff radii for the Coulomb and Lennard-Jones interactions are $r_{c, Coul} = {10}{\text{\AA} }$ and $r_{c, LJ}= {12}{\text{\AA} }$, respectively. The long-range electrostatic interactions are treated using the Particle–Particle–Particle-Mesh algorithm^42^ with an accuracy of $10^{-5}$. The SHAKE algorithm controls the bond lengths and angles for rigid molecules. The integration time step is 2 fs. The length of molecular dynamics trajectories is from 1 ns to 100 ns. The calculations are performed in *NVT* (the Nose-Hoover thermostat) and *NPT* ensembles (the Nose-Hoover barostat). Visualization and analysis are carried out with Ovito^43^.

### Elastic moduli

Suzuki et. al^44^ note that it happens that for the same material, different values of elastic moduli are obtained, depending on the methods used to calculated them. In addition, it may turn out that the same method is in good agreement with experimental data for substances with a structure of one type, but for another structure it already poorly reproduces what is obtained in practice. This clearly demonstrates the limits of applicability of one method or another. Meanwhile, it is important how well the model used describes the properties of a real system, in comparison with alternative ones, especially for water, for which more than a hundred different models have been proposed, but each is used for specific purposes^26^. Unlike other works on molecular dynamics modelling^44,45^, which study elastic properties with special emphasis on structural phase transitions, where elastic moduli are calculated using the fluctuation formula of molecular dynamics, here we calculate the elastic moduli directly^46^.

The definition of the isothermal bulk modulus of elasticity is given by:

$$\begin{aligned} B=-V\left( \frac{\partial P}{\partial V}\right) _T=\rho \left( \frac{\partial P}{\partial \rho }\right) _T, \ \end{aligned}$$

where *P* is the pressure, *V* is the volume, $\rho$ is the density of the system. To find the bulk modulus, after equilibration in the *NVT* ensemble for at least 2 ns, it is necessary to compress the simulation box by changing the linear dimensions by $\pm 1\%$ (while the deformation is linear) in each of the three directions and find the slope coefficient $P(\rho )$.

The shear modulus is by definition:

$$\begin{aligned} G=-\frac{d\tau _{xy}}{d\gamma _{xy}}, \end{aligned}$$

where $\tau _ {xy}$ is the shear stress, $\gamma _{xy}$ is the shear strain.

To calculate the shear modulus *G*, by analogy with the bulk modulus *B*, we carry out a shear strain of $\pm 1-2\%$ in one direction. *G* is found as the slope coefficient $\tau (\gamma )$. It is important that our system is isotropic with respect to the shear modulus. We have carried out three independent deformations in different directions and made sure that the result does not depend on the direction of the shear (see Supplementary Fig. S1 online). The values of the elastic moduli for each state (*P*, *T*) are obtained by averaging over 3–5 independent straining simulations.

**Figure 2:**
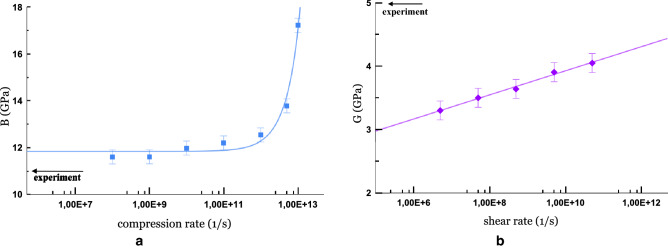
The dependence of elastic moduli of HDA on the relative strain rate for small deformation. (**a**) The compression rate dependence of the bulk modulus at $T\,=\,77\,\hbox {K}$ and $P\,=\,0.17\,\hbox {GPa}$; the solid blue line shows an exponential interpolation. (**b**) The shear rate dependence of the shear modulus at $T\,=\,77\,\hbox {K}$ and $P\,=\,0.05\,\hbox {GPa}$; the solid purple line shows a logarithmic interpolation. The abscissa axis is in the logarithmic scale. The experimental values^30^ are shown by arrows.

In addition, the strain rate can be varied, but this affects the values of the elastic moduli. Fig. 2 shows the values of *B* and *G* for calculations with different rates of relative deformation under the same conditions. For the bulk modulus (Fig. 2a), exponential convergence is observed with decreasing compression rate. Moreover, for lower velocities, the value is closer to the experimental one^30^ $B_ {exp}\,=\,11\,\hbox {GPa}$, where the elastic moduli are found by the ultrasonic method at frequencies of 5 MHz. Actually, the ultrasonic method involves the measurement of adiabatic characteristics, so that the slight difference in values is probably due to the fact that we consider the isothermal modulus. Thus, there is not much benefit in using very low compression rates (less than $10^9\,\hbox {s}^{-1}$). The behavior of the strain rate dependence of the shear modulus is different. No convergence is observed in this case, and there is a significant discrepancy (about 30%) with the experimental value $G_{exp}\,=\,5\,\hbox {GPa}$. Further, shear modulus calculations are carried out at a shear deformation rate of $5\cdot 10^7\,\hbox {s}^{-1}$.

We are able to calculate the bulk modulus of amorphous ice with a deviation of less than 10% from the experimental value, while for hexagonal ice we have a deviation of about 70%, this is consistent with the work Moreira et al.^47^. They took a similar approach using several water models TIP4P/Ice including.

## Preparatory stage

### Preparation of LDA

**Figure 3:**
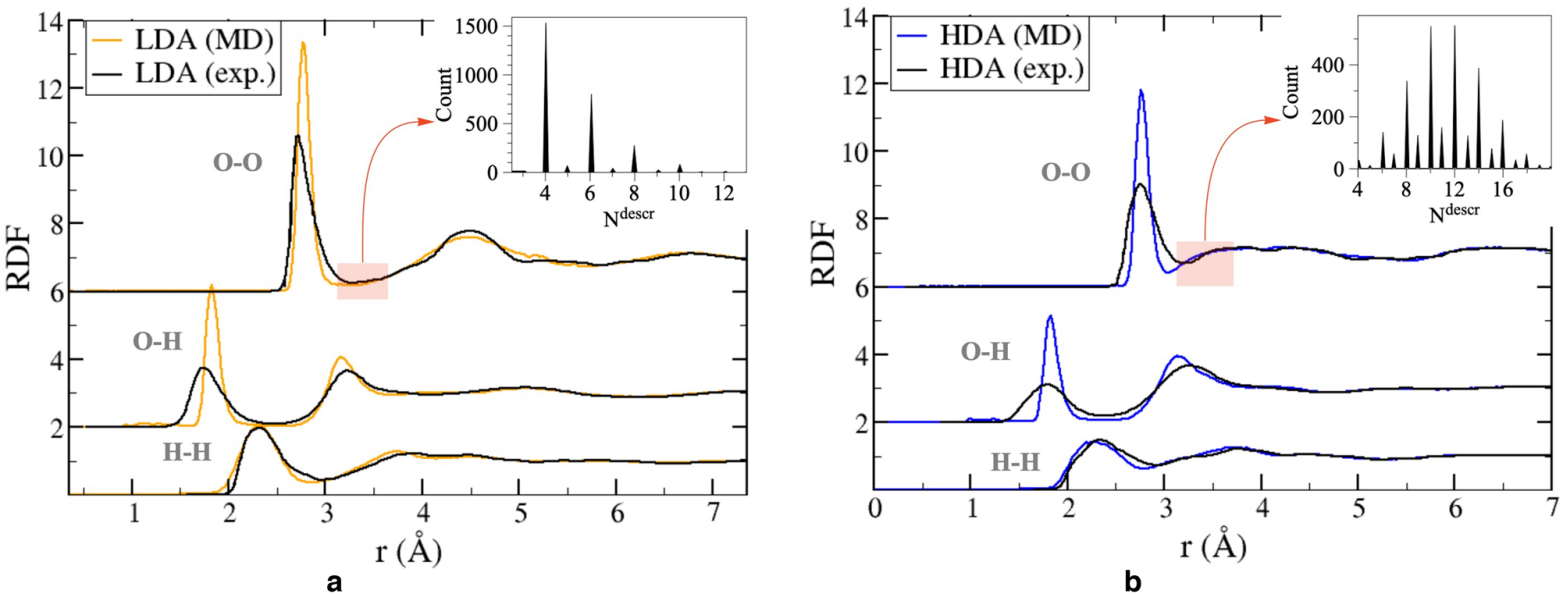
The radial distribution functions of LDA (**a**) and HDA (**b**) at *T* = 77 K and *P* = 0.1 MPa for TIP4P/Ice water model and the experimental data^31^. The corresponding distributions of oxygen atoms with respect to $N^{descr}$ (the number of neighbours in the spherical layer $3.1< r < 3.7$ Å) are given in the upper right corners of (**a**) and (**b**).

In general, water is a poor glass former, and in practice a cooling rate of more than 0.01 K/ns is required to avoid crystallization. We obtain LDA by fast isobaric cooling of liquid water at a rate of 10 K/ns. It was also shown^48^ that the structure of the resulting amorphous ice changes slightly when the cooling rate changes by 1–2 orders of magnitude, and the density deviations are within the statistical error. As a result, we get a system at $T\,=\,77\,\hbox {K}$ and $P\,=\,0.1\,\hbox {MPa}$. with a density of $0.95\,\hbox {g/cm}^3$, whereas in the experiment under such conditions the density is $0.94\,\hbox {g/cm}^3$. As can be seen in Fig. 3, the radial distribution function is in good qualitative agreement with the experimental one^31^, but the first peak is overestimated, which was already shown earlier^36^ for the TIP4P/Ice liquid state.

### Preparation of HDA

To obtain HDA, hexagonal ice at a temperature of 77 K and a pressure of 0.1 MPa is isothermally compressed (NVT ensemble, compression is performed by decreasing the size of the computational cell linearly with time) from a density of 0.95–1.31 g/cm^3^. After equilibration the system pressure is 1.4 GPa. Upon further release of the pressure 0.01 MPa, the amorphous structure is retained, the system remains metastable. An amorphous form is actually obtained that differs from LDA, its density at $T\,=\,77\,\hbox {K}$ and $P\,=\,0.1\,\hbox {MPa}$ is $1.15\,\hbox {g/cm}^3$. Moreover, RDF of HDA has noticeable differences (Fig. 3). The second HDA’s peak has a broader shape and a slight splitting at 4 Å. Also, there is an increased probability of finding water molecules at an O-O distance of 3.1–3.7 Å from the central water molecule. We repeated the calculation for a larger system, but no significant size effect is observed (see Supplementary Fig. S2 online).

In the experiment^5^, SSA of hexagonal ice occurs already at 1.1 GPa, but the compression rate plays a certain role. In the simulation, we consider isothermal compression with different compression rates in the range of 0.1-5 GPa/ns. In Supplementary Fig. S2 online dependencies of $P(\rho )$ for different compression rates are given. The amorphization pressure decreases with decreasing compression rate. At the same time, our lowest compression rate $q\,=\,0.1\,\hbox {GPa/ns}$ (in terms of density, this corresponds to a compression rate of $0.05\,\hbox {g/cm}^3/\hbox {ns}$), which is several orders of magnitude higher than the experimental rate^30^ $10^{-12}\,\hbox {GPa/ns}$. It is problematic to mimic the compression rate in molecular dynamics as in the experiment, nevertheless, the amorphization pressure obtained by us in the MD model is extrapolated to the corresponding experimental value well .

## Results

### Pressure-induced transformations

**Figure 4:**
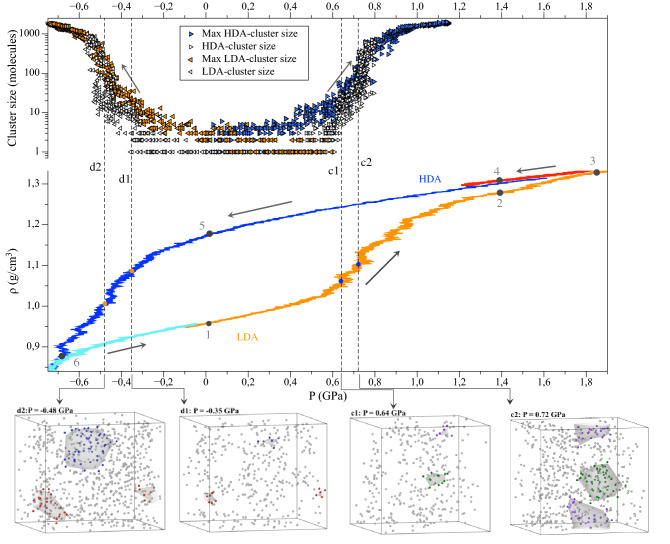
The pressure dependence of density during the transformations of amorphous ices under pressure at $T\,=\,77\,\hbox {K}$, the compression rate is 0.1 GPa/ns. The arrows indicate the cycle direction. On the route $4\rightarrow 5$, the blue line is the decompression of HDA obtained during SSA from Ih, the red line $3\rightarrow 4$ is the decompression of HDA obtained from LDA. The cyan line $6\rightarrow 1$ is the compression of LDA obtained from HDA. In the upper part of the plot, there is the pressure dependence of the size of the largest cluster defined according to our selection rules with the thresholds $N_{descr} \leqslant 5$ for LDA and $N_{descr} \geqslant 11$ for HDA: the blue right triangles show the maximum HDA-cluster size during the compression of LDA and the orange left triangles show the maximum LDA-cluster size during the decompression of HDA; the corresponding open right (left) triangles show the size of one selected smaller HDA (LDA) cluster. Below, the snapshots c1 and c2 demonstrate two HDA-clusters (their oxygen atoms are highlighted in green and purple) at pressures of 0.64 GPa and 0.72 GPa. The snapshots d1 and d2 demonstrate two LDA-clusters (their oxygen atoms are highlighted in blue and red) at pressures of −0.35 GPa and −0.48 GPa. Molecules with the same indices present in the clusters of one color that is why the snapshots illustrate the growth of clusters with time.

In this section of our study, we carry out isothermal ($T\,=\,77\,\hbox {K}$) compression/decompression of amorphous ices. For the initial system, we take the LDA obtained earlier (point 1 in Fig. 4) and compress it at a rate of 0.1 GPa/ns to a pressure of 2 GPa (point 3). Then the pressure is gradually released, until negative values (point 6), and compression begins again. Thus, in our model, transformations between amorphous forms of ice occur in a cycle that resembles “hysteresis”, as in experiments^30,49^.

In fact, in MD we have implemented two methods for obtaining HDA: from hexagonal ice and from LDA (point 4 in Fig. 4). Comparing the radial distribution functions of the resulting structures (see Supplementary Fig. S3 online), we can conclude that the HDA structure is independent of the method of its formation for these two cases considered.

**Figure 5:**
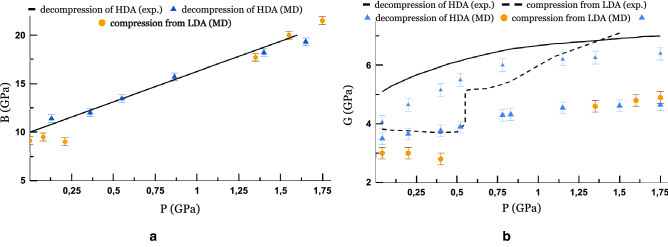
The pressure dependence of elastic moduli during isothermal ($T\,=\,77\,\hbox {K}$) compression and decompression at a compression rate 0.1 GPa/ns: (**a**) the bulk modulus, (**b**) the shear modulus. The dotted line corresponds to the experimental data during LDA compression at 110 K. The small blue symbols correspond to the results of the calculation with a higher shear rate of $5\cdot 10^{10}\,\hbox {s}^{-1}$ during decompression of HDA.

By analogy with a conventional phase transition, the transition from LDA to HDA implies a change in local structure and should lead to a change of elastic characteristics. The dependence of the bulk modulus of elasticity on pressure during HDA decompression is shown in Fig. 5a. We also calculate the bulk modulus of LDA at the beginning and at the end of the compression, but this method does not allow finding intermediate values, since at 0.5–1.5 GPa the system is unstable. Nevertheless, one can be convinced that upon compression of LDA, the elastic modulus increases to values similar to HDA. During HDA decompression, the result of our model is in good agreement with the experiment^30^ for the pressure dependence of bulk modulus. For the shear modulus, we have a slightly larger quantitative difference (Fig. 5b).

### Heating-induced transformations

Before we start discussing isobaric heating of amorphous ices, it is necessary to understand what rate of heating makes it possible to obtain an adequate description at a not very high computational costs. To do this, we carry out several processes of HDA heating at constant pressure (0.05 GPa) at different rates in the range $Q_{heat}\,=\,0.25\hbox {-}50\,\hbox {K/ns}$, and Fig. 4.2a shows how the density behaves with increasing temperature. A heating rate of 7 K/ns already allows us to observe a characteristic change in density, and this is a good compromise with computational cost, so we settle on this value.

**Figure 6:**
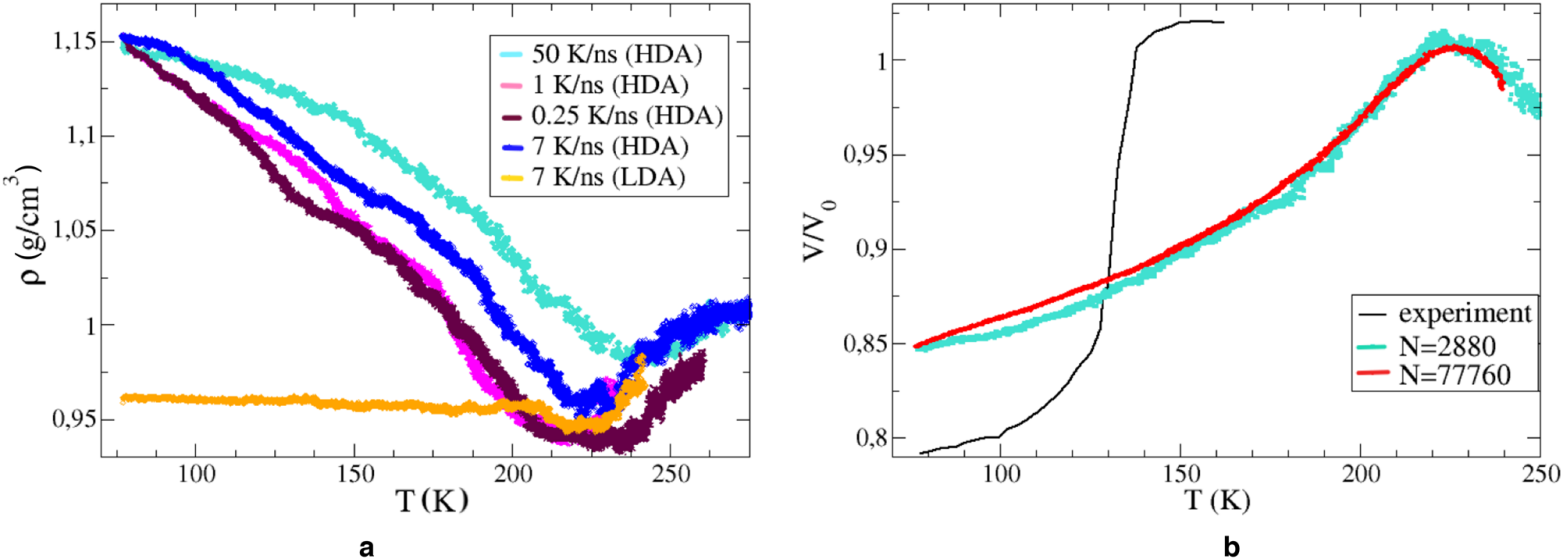
(**a**) The temperature dependencies of density during the isobaric heating of HDA for several heating rates. The orange line shows the dependence in the process of heating of LDA at the rate of 7 K/ns. (**b**) The absence of the size effect during isobaric (*P* = 0.05 GPa) heating of HDA. The MD-heating rate is 7 K/ns; the experiment^30^ assumes much slower heating.

Thus, the temperature dependencies of density of LDA and HDA under isobaric heating at the same rate is shown in Fig. 4.2a. And as it is not difficult to see, at $T\,>\,210\,\hbox {K}$ the densities of the systems become almost the same. Moreover, at a temperature of 240 K, the structures of heated LDA and HDA are identical, which is indicated by the similarity of the RDF and the distribution of angles between oxygen atoms (see Supplementary Fig. S5 online), that is, we can say that the transformation of LDA and HDA into liquid water has occurred.

A more interesting question is what happens to amorphous ices before the transition to the liquid water phase? Trying answering this question, we compare the structure of the systems at 77 K and 180 K. How the RDF and the tetrahedral structure change can be seen in more detail in Supplementary Fig. S5 online. Up to 180 K, an increase in the peak of the angular distribution is observed, from which it can be concluded that, before the liquid phase, the HDA passes into an LDA-like state. Since the RDF is an ensemble-averaged characteristic of the system, the intermediate states between 90 and 180 K are more likely to relate to a mixture of two states, where HDA first prevails, and then LDA.

**Figure 7:**
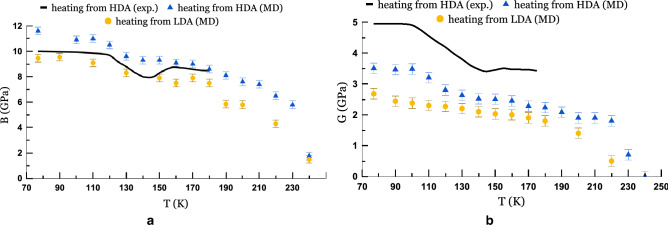
The temperature dependencies of (**a**) the bulk modulus and (**b**) the shear modulus during isobaric heating ($P\,=\,0.05\,\hbox {GPa}$).

It should be mentioned that under the experimental conditions at HDA heating, a rather sharp jump of 15% in the density (or specific volume) occurs at a temperature of 130–140 K^30^, which was attributed by authors precisely to the HDA-LDA transition. In our modelling, however, the specific volume increases gradually (Fig. 4.2b). This fact is can be associated with both the high heating rate and the use of the barostat, which facilitates the processes of local structural rearrangements. Besides, the behavior of the temperature dependencies of specific volume for systems with 2880 and 77760 molecules is practically the same.

As for the temperature dependencies of the elastic moduli (Fig. 7), we obtain a fairly good quantitative agreement with experiment^30^ for the bulk modulus; however, we do not observe qualitatively no-monotony behavior in the model, as well as crystallization into cubic ice at 160 K with which these peculiarities are associated. The calculation of the shear modulus does not give such a good quantitative agreement. Nevertheless, both characteristics reflect softening of the amorphous network of LDA and HDA. The relatively sharp decrease in the bulk modulus of LDA at 190 K is most likely precedes further transformation into liquid water. And the decrease in the shear modulus at a temperatures above 225 K to practically zero values is consistent with the fact that both systems transform into the liquid phase.

### Formation of new phase clusters during LDA $\leftrightarrow$ HDA transitions

The density jump and the hysteresis are characteristic features of first-order phase transitions that are accompanied by nucleation. The similarity of LDA $\leftrightarrow$ HDA transformations with first-order phase transitions is a long-standing topic of debates both in theoretical and in experimental studies (e.g., see^12,21^). But then one should expect that a process analogous to nucleation takes place during LDA $\leftrightarrow$ HDA transitions. Using the results of our MD calculations, we make an attempt to describe the mechanism of the formation of HDA clusters during the compression of LDA and the formation of LDA clusters the reverse process.

The selection rules for new phase detection plays a crucial role in such an analysis. In this work we use quite a simple algorithm based on the difference of RDFs for LDA and HDA structures. We use the fact that in HDA phase for an oxygen atom, the probability of detecting a neighbouring oxygen atom at a distance of 3.1–3.7 Å increases (Fig. 3). Let us denote the number of neighbours in the spherical layer $3.1< r < 3.7$ Å as $N^{descr}$. The algorithm for detection of HDA/LDA clusters works as follows:

1. $N^{descr}$ is calculated for each oxygen atom.
2. If $N^{descr}\geqslant \,11$, then the atom corresponds to the HDA structure. If $N^{descr} \leqslant 5$, then the atom corresponds to the LDA structure. Molecules with intermediate values $6\leqslant N^{descr}\leqslant 10$ are excluded from the analysis. We will refer to this selection rule as the more stringent criteria (the less stringent criteria will be introduced below).
3. An HDA/LDA-atom *i* is included in a cluster if there is an HDA/LDA-atom *j* and $|r_i-r_j| < 3.0$ Å.

The upper part of Fig. 4 shows the results of the application of the proposed selection algotithm. First, in the process of the LDA $\rightarrow$ HDA transition under isothermal compression ($T\,=\,77\,\hbox {K}$), the number of particles characteristic of HDA increases with increasing pressure; in addition, growing HDA domains are formed, which is shown in Fig. 4 (see also Supplementary Fig. S6 online). At a pressure of about 0.6 GPa a noticeable growth of domains begins. And the size of the smallest such cluster that accompanies intensive growth can be visually estimated as 5–20 molecules (this corresponds to a spherical domain of 4–9 Å). The transition pressure can be taken as 0.74 GPa, at which cluster growth is the most intense (we mark the corresponding value on the T-P diagram (Fig. 1). By analogy with the previous case, in the inverse transformation HDA $\rightarrow$ LDA during isothermal decompression formation of growing LDA clusters is observed as well. This supports the experiment of Tonauer et al.^50^, in which the HDA $\rightarrow$ LDA transformation during decompression at 140 K was considered and the “nucleation” phenomenon was revealed. The radius of a (spherical) LDA seed was estimated as 3–8 Å^50^.

The intermediate values $6 \leqslant N^{descr} \leqslant 10$ introduce a certain ambiguity to the selection algorithm considered. Following Fig. 3, molecules in both the LDA-like and the HDA-like local environments can have $N^{descr}$ in this range. In order to address the sensitivity of the algorithm to the choice of the threshold values, we consider below the case of $N^{descr}\geqslant \,10$ for HDA and $N^{descr} \leqslant 6$ for LDA molecules.

**Figure 8:**
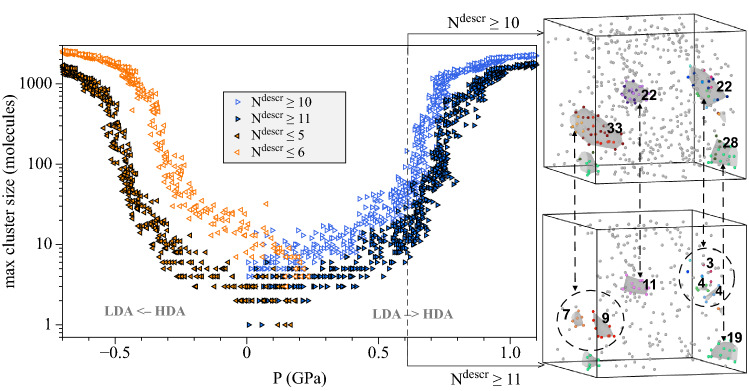
Pressure-induced LDA $\leftrightarrow$ HDA transformations at $T\,=\,77\,\hbox {K}$. The pressure dependence of the size of the largest cluster defined according to our algorithm for two variants of the threshold values of $N^{descr}$ for LDA/HDA selection. Two snapshots show several HDA clusters at the moment corresponding to $P\,=\,0.61\,\hbox {GPa}$. The lower snapshot shows the oxygen atoms with $N^{descr} \geqslant 11$ (the largest cluster has 19 molecules), seven HDA-clusters are highlighted by the color and the surface mesh (a number next to a cluster indicates how many molecules it contains). The top snapshot for the same time moment shows the oxygen atoms with $N^{descr} \geqslant 10$ (the largest cluster has 33 molecules) and the HDA-clusters that includes the clusters shown on the lower snapshot (additional atoms are highlighted in dark color).

On the one hand, the less stringent selection rule ($N^{descr}\geqslant \,10$ for HDA and $N^{descr} \leqslant 6$ for LDA molecules) means that more particles are involved in the analysis of cluster formation. On the other hand, the more stringent criteria ($N^{descr}\geqslant \,11$ for HDA and $N^{descr} \leqslant 5$ for LDA molecules) makes it possible to assign a molecule to one or another structure with a higher probability. As can be seen from the distributions in Fig. 3, LDA has a small fraction (about 3%) of molecules with $N^{descr}\,=\,10$, and HDA has a small fraction (about 5%) molecules with $N^{descr}\,=\,6$.

The following differences between the less stringent and the more stringent criteria can be mentioned:

1. In the less stringent case the “unassigned” molecules with $7 \leqslant N^{descr} \leqslant 9$ form small clusters, but in the more stringent case the molecules with $6 \leqslant N^{descr} \leqslant 10$ form a common background (see Supplementary Fig. S7 online).
2. In the less stringent case the number of molecules in the new phase clusters is higher (see Fig. 8). This effect is more pronounced for LDA clusters in HDA under decompression. For example, the snapshots on Fig. 8 show how the number of molecules of HDA clusters increases when the selection rule is changed from the more stringent variant to the less stringent variant. Clusters selected via $N^{descr} \geqslant 10$ are bigger and some big clusters are results of merging several small clusters selected via $N^{descr} \geqslant 11$. However, the increase in volume of each cluster is less pronounced that the increase in its number of molecules.
3. In the more stringent case the new phase clusters vanish to 1–4 molecules close to the hypothetical LDA-HDA equilibrium where metastability disappears. In the less stringent case the clusters of $\sim 10$ HDA-like molecules under LDA compression and $\sim 20$ LDA-like molecules for HDA decompression are observed. This observation suggests that the more stringent selection rule suits better for the detection of the new phase formation (that is why we use for the kinetic spinodal shown on Fig. 1).

During isobaric heating ($P\,=\,0.05\,\hbox {GPa}$) of HDA, the number of LDA clusters gradually increases without the evident growth of domains (see the variation of the selected LDA-cluster in Supplementary Fig. S8 online). Therefore, in this case, we see fluctuations-based growth of LDA state, and there are no arguments to speak about a cluster growth (nucleation-like) process. In order to consider possible system size effects, we perform the same modelling for the system of 77760 molecules, but the nature of the transition remains qualitatively the same without any visible cluster growth features. The absence of the formation of clusters that trigger growth events during this isobaric heating can be explained by the nearly constant level of metastability along the isobar that goes parallel to the LDA-HDA equilibrium line.

## Discussion

In this work using the MD modelling with the TIP4P/ice potential we have confirmed that the model gives a consistent description of LDA and HDA amorphous ices and have reproduced the key experimental results on LDA $\leftrightarrow$ HDA transitions^30^:

1. There is a satisfactory agreement with experimental results^31^ for the RDFs of LDA and HDA. The extra height of the first peaks of RDFs in the TIP4P/Ice molecular dynamics tells that the O–O and H–O bonds are too rigid. This fact, however, is a well-known feature of classical water models that do not take into account nuclear quantum effects at low temperatures^51,52^.
2. During the isothermal SSA of Ih into HDA, the amorphization pressure depends on the compression rate, but no significant size effect was observed. The MD results at varying (very high) compression rates show a reasonable convergence to the experimental results.
3. The non-equilibrium modelling of pressure-induced transformations between LDA and HDA reflects the main features observed experimentally: the jump in density at the LDA $\rightarrow$ HDA transition and the hysteresis. The reverse transition of the HDA $\rightarrow$ LDA during decompression is observed at negative pressures. The RDFs of HDA structures obtained from Ih and from LDA are indistinguishable.
4. We have analyzed the compression rate and share rate dependencies in the MD calculations of elastic moduli. We show that the bulk modulus converges quickly at decreasing compression rate. However, no convergence has been detected for the shear modulus at decreasing shear rate. Therefore, the calculations have been performed with the shear rate close to the corresponding ultrasonic frequency in the experiment^30^. The results of the corresponding MD calculations of bulk and shear moduli of HDA are in a good overall agreement with the experimental data^30^ for the case of isothermal compression and for the case of isobaric heating. There is a very good quantitative agreement for the bulk modulus (the accurate prediction of the liquid water bulk modulus at room temperatures in TIP4P/2005 model has been reported recently as well^23,53^). However, the MD results for the shear modulus are about 50% lower that the experimental data. Here it is worth mentioning that the agreement for the TIP4P/Ice result for the bulk modulus of Ih is about 70% higher that the experimental value (similarly as it was shown previously^47^). It has been shown^54,55^ that including nuclear quantum effects in MD modelling improves the prediction of the Ih bulk modulus.

5.1 In the framework of the presented model, we have developed a nearest neighbours analysis method that distinguishes clusters of HDA in LDA and vice versa. Using this method, we have shown that both the LDA $\rightarrow$ HDA and the HDA $\rightarrow$ LDA transformations at isothermal compression/decompression proceed via the formation and growth of new phase clusters as soon as a high degree of metastability is achieved. There are clear pictures of domains formation and growth events in the system of 2880 molecules for both transitions. Fig. 4 shows that at the pressure of thermodynamic LDA-HDA equilibrium there are clusters of the competing structure formed by 1–4 molecules. These clusters can be regarded as local “subcritical” fluctuations. In the region of higher metastability the size of such cluster increases. The mechanism of their growth and thermodynamic analysis requires a separate study. Here we want to emphasize that the new amorphous structure (LDA in HDA and vice versa) form as localized domains. We can call a kinetic spinodal the narrow range of pressures when the size of a larges cluster grows rapidly at compression of LDA (or decompression of HDA). These pressures obtained in our calculations for four temperatures are shown on Fig. 1 as a boundary of kinetic stability of the respective amorphous ices (and supercooled water at temperatures close to LLCP). The location of such a kinetic spinodal should depend on the compression/decompression rate. We can notice that the shape of a non-equilibrium kinetic spinodal obtained in this study are in a reasonable agreement with the LLCP parameters determined recently for TIP4P/Ice model^17^.

5.2 Both the more stringent and the less stringent variants of the $N^{descr}$ threshold values used for LDA/HDA detection show that the transition between LDA and HDA proceeds via formation of localised clusters of new phase. In the amorphous solids as we consider and at low temperatures any diffusion of molecules is practically not possible. We observe two mechanisms of growth of these new phase clusters: the attachment of individual molecules and the merging of initially separated clusters. Hence, on the basis of our observations we can say that the formation of new phase in the LDA $\leftrightarrow$ HDA transformations has evident aspects of the nucleation process typical to first-order transitions.

5.3 The most problematic case for the MD modelling considered is the isobaric heating of HDA. In this, case we observe in MD modelling a gradual transition of HDA to LDA-like state and then to liquid water. Contrary to the experimental data, in MD modelling there is no rapid increase of specific volume. The absence of the formation of clusters that trigger growth events can be explained by the nearly constant level of metastability during this isobaric heating process: the system moves on the T-P diagram in parallel to the LDA-HDA equilibrium line. Moreover, the system approaches the region near the second critical point^17^. It is known that the size of critical nucleus becomes larger when the system approaches its critical point. Therefore, we can make a hypothesis that the proper explanation of the experimental data on the isobaric heating requires a theory for HDA-LDA-liquid water transformations at larger time and length scales well beyond the MD scale.

## Conclusions

The TIP4P/Ice molecular dynamics modelling of amorphous ices and LDA $\leftrightarrow$ HDA transformations gives consistent results that have been compared with the ultrasonic measurements of elastic constants in HDA, LDA and during the LDA $\rightarrow$ HDA isothermal transformation and during the HDA $\rightarrow$ LDA isobaric transformation by Gromnitskaya et al.^30^ MD modelling gives the correct qualitative description of the pressure and temperature dependencies of bulk and shear moduli of HDA and LDA in these processes. The TIP4P/Ice water model provides a quantitatively accurate description for the HDA bulk modulus, but for the HDA shear modulus the absolute values are significantly lower than the experimental values.

Molecular dynamics modelling captures the essential features of the LDA $\leftrightarrow$ HDA transition observed experimentally. Therefore, the model is able to supplement the experiment and allows studying in details the reverse process of HDA $\rightarrow$ LDA isothermal transformation that takes place at negative pressures. Using this model, we have shown that both types of isothermal LDA $\leftrightarrow$ HDA transformations proceed via the mechanism of formation of new phase clusters at high levels of metastability.

## Supplementary Information

Supplementary Information 1.

Supplementary Information 2.

## Data Availability

We provide a link to the most significant data that illustrate the observed “nucleation” events during isothermal LDA $\leftrightarrow$ HDA transitions https://github.com/garkulic/Nucleation-during-the-LDA-HDA-transition. The rest of the data requires a deep dive into the analysis process. We are ready to provide other information to the interested readers on a reasonable request.
