# Supplementary material for: Molecular dynamics analysis of elastic properties and new phase formation during amorphous ices transformations
Source: Sci Rep. 2022 Aug 3;12:13325. doi: 10.1038/s41598-022-17666-2 (PMC9349219; doi:10.1038/s41598-022-17666-2)
Supplement: Supplementary file 1 — Supplementary Information 1. [file 41598_2022_17666_MOESM1_ESM.pdf]

Molecular dynamics analysis of elastic properties and new phase formation during amorphous ices transformations (Supplementary information)

Anastasiia Garkul[1,2,*] **and Vladimir Stegailov**[1,2,3]

[1]Joint Institute for High Temperatures of the Russian Academy of Sciences, 125412 Moscow, Russia
[2]Moscow Institute of Physics and Technology (National Research University), 141701 Dolgoprudny, Russia
[3]National Research University Higher School of Economics, 109028 Moscow, Russia
[*]garkul.aa@phystech.edu

Supplementary video file

Video 1. See the file 'LDA-cluster.mov". The video shows the process of growth of one selected LDA-cluster (highlighted in green) in the transformation of HDA → LDA during isothermal decompression (T = 77 K) in the pressure region from -0.2 to -0.5 GPa. The cluster is determined in accordance with the algorithm described in our article. The alpha-shape algorithm in Ovito was used for constructing the dividing surface. The remaining oxygen atoms with the LDA-like local environment (grey particles) appear on the video over time. The total number of molecules in the system is 23040.

Supplementary figures

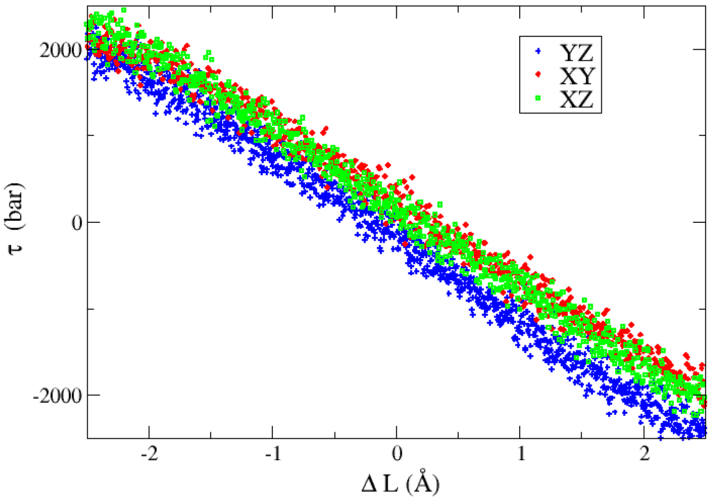

Figure S1. The shear deformation of HDA in different directions at T = 77 K and P = 0.05 GPa. Strain rate is $5 \cdot 10^{-6}$ fs^{-1}.

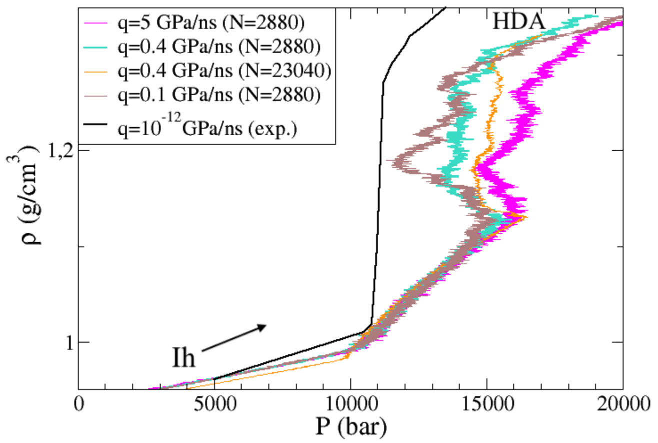

Figure S2. The pressure dependencies of density during isothermal (T = 77 K) compression of Ih-ice with several compression rates and for N = 2880 and N = 23040 molecules.

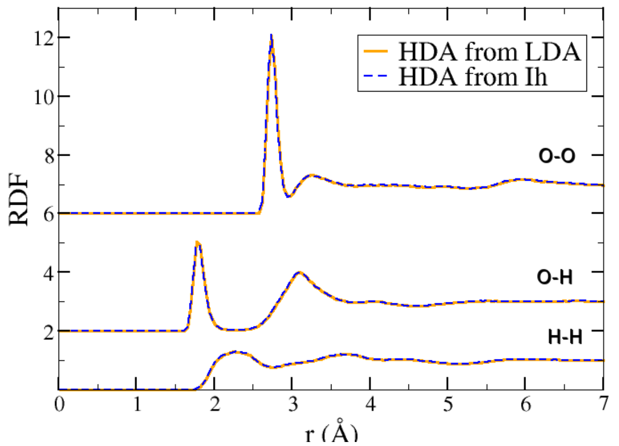

Figure S3. The comparison of the partial RDFs of HDA obtained by SSA from Ih and by compression from LDA, at T = 77 K and P = 1.4 GPa

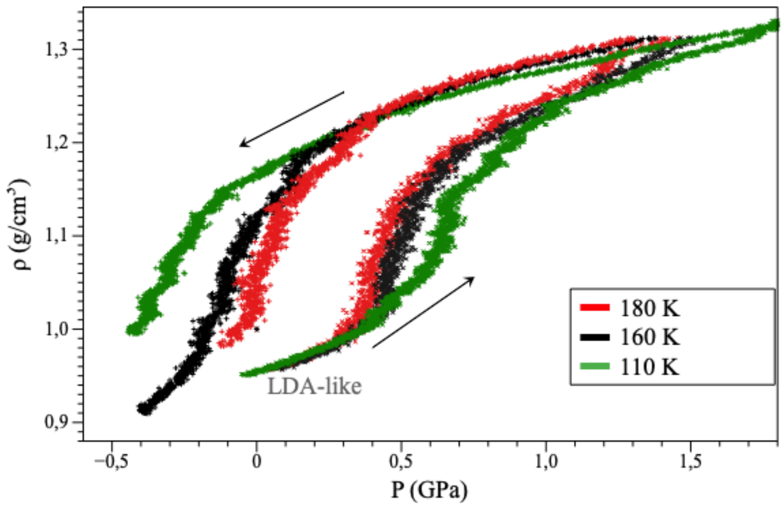

Figure S4. The pressure dependencies of density during isothermal compression of LDA-like system and subsequent decompression for different temperatures. The compression rate is 0.1 GPa/ns.

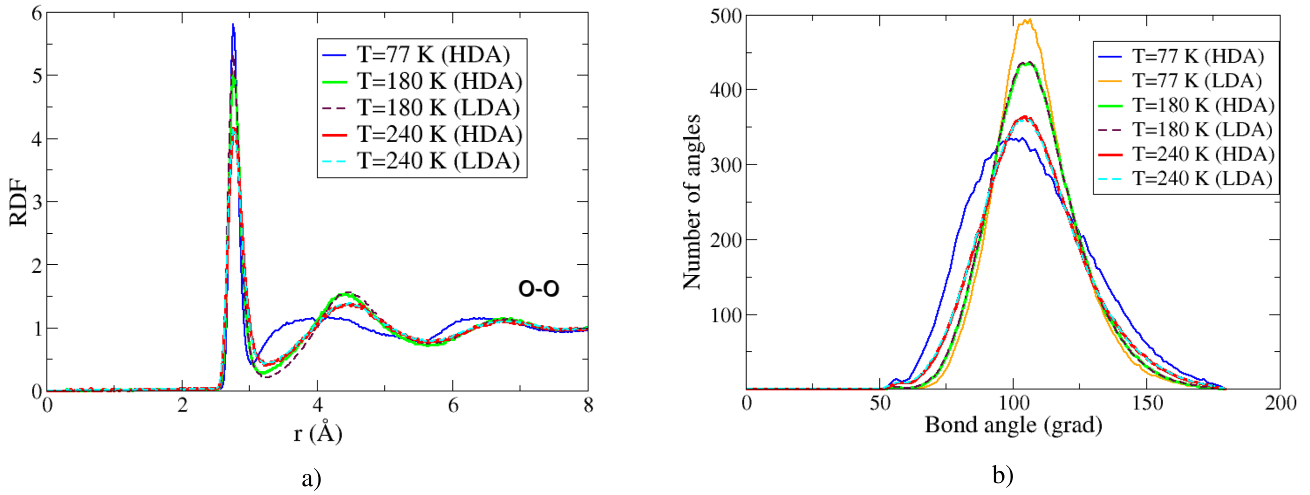

Figure S5. The structural change upon the isobaric heating of HDA. a) The RDFs O-O; b) The distribution of bond angles formed by pairs of bonds meeting at the same oxygen atoms.
The initial structure is indicated in the brackets.

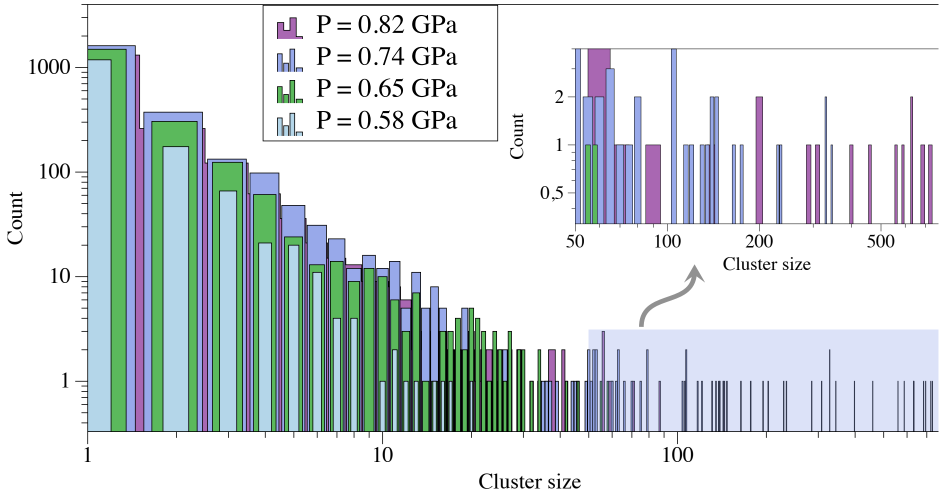

Figure S6. The distribution of HDA-clusters by their size for four moments of the LDA compression process during at the corresponding pressures.

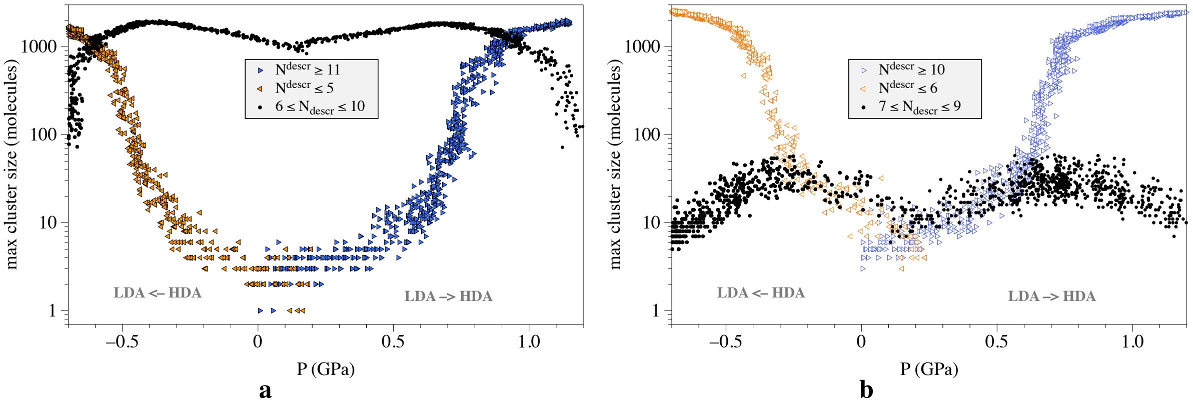

Figure S7. The pressure dependence of the size of the largest cluster defined according to our algorithm for different choice of N^{descr}.

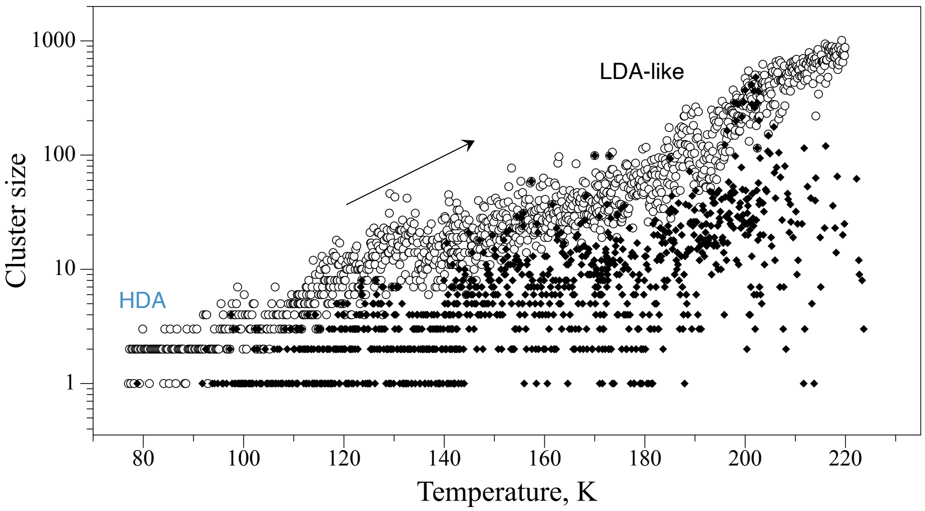

Figure S8. The temperature dependencies of the size of the largest LDA-cluster (open symbols) and the size of one selected LDA-cluster (black solid symbols) during isobaric heating (*P* = 0.05 GPa) of HDA. Critical phenomena are not observed.
